# Histone deacetylase inhibition by Entinostat for the prevention of electrical and structural remodeling in heart failure

**DOI:** 10.1186/s40360-019-0294-x

**Published:** 2019-03-06

**Authors:** Johanna K. Freundt, Gerrit Frommeyer, Tilmann Spieker, Fabian Wötzel, Jochen Schulze Grotthoff, Jörg Stypmann, Georg Hempel, Michael Schäfers, Andreas H. Jacobs, Lars Eckardt, Philipp S. Lange

**Affiliations:** 1 Department of Cardiology II: Electrophysiology, University Hospital Münster, Münster, Germany; 2 Department of Pathology, University Hospital Münster, Münster, Germany; 3 Department of Cardiovascular Medicine, University Hospital Münster, Münster, Germany; 4 Institute for Pharmaceutical and Medical Chemistry, University of Münster, Münster, Germany; 5 European Institute for Molecular Imaging, University Hospital Münster, Münster, Germany; 6 Department of Nuclear Medicine, University Hospital Münster, Münster, Germany

**Keywords:** HDAC, Entinostat, Arrhythmias, Remodeling, Fibrosis

## Abstract

**Background:**

The development of heart failure is accompanied by complex changes in cardiac electrophysiology and functional properties of cardiomyocytes and fibroblasts. Histone deacetylase (HDAC) inhibitors hold great promise for the pharmaceutical therapy of several malignant diseases. Here, we describe novel effects of the class I HDAC inhibitor Entinostat on electrical and structural remodeling in an in vivo model of pacing induced heart failure.

**Methods:**

Rabbits were implanted a pacemaker system, subjected to rapid ventricular pacing and treated with Entinostat or placebo, respectively. Following stimulation, rabbit hearts were explanted and subsequently subjected to electrophysiological studies and further immunohistological analyses of left ventricles.

**Results:**

In vivo, rapid ventricular stimulation caused a significant prolongation of monophasic action potential duration compared to sham hearts (from 173 ± 26 ms to 250 ± 41 ms; cycle length 900 ms; *p* < 0.05) and an increased incidence of Early afterdepolarisations (+ 150%), while treatment with Entinostat in failing hearts could partially prevent this effect (from 250 ± 41 ms to 170 ± 53 ms, *p* < 0.05; reduction in EAD by 50%). Entinostat treatment partially restored KCNH2 and Cav1.3 gene expressions in failing hearts, and inhibited the development of cardiac fibrosis in vivo.

**Conclusion:**

In a rabbit model of heart failure, Entinostat diminishes heart failure related prolongation of repolarization and partially restores KCNH2 and Cav1.3 expression. In addition, Entinostat exerts antifibrotic properties both in vitro and in vivo. Thus, Entinostat might be an interesting candidate for the pharmaceutical therapy of heart failure directed against structural and electrical remodeling.

**Electronic supplementary material:**

The online version of this article (10.1186/s40360-019-0294-x) contains supplementary material, which is available to authorized users.

## Background

Ventricular tachyarrhythmias are an important predictor of morbidity and mortality in patients with chronic heart failure [1]. Moreover, sustained tachyarrhythmia can lead to ventricular remodeling impairing left ventricular ejection fraction thereby causing a tachycardiomyopathy. Despite a significant progress in pharmacological and interventional antiarrhythmic therapies, treatment options for patients suffering from heart failure are still limited. The development of heart failure is accompanied by complex changes in cardiac electrophysiology and functional properties of cardiomyocytes and fibroblasts, most of which are still poorly characterized. Given the multitude of cell types and signaling cascades that are involved in the process of ventricular remodeling, effective therapeutic strategies would ideally simultaneously impact several different remodeling pathways in the diverse cell types involved [2]. Histone deacetylases (HDACs) may represent such targets. HDACs play a central role in epigenetic regulation of transcription and cell proliferation [3, 4] and in the context of a whole host of key biological processes, including pathological remodeling of the heart [5]. Reversible histone acetylation provides a central mechanism for controlling gene expression and cellular signaling events in multiple cells types, e.g., myocytes, fibroblasts and immune cells [6, 7]. Over the last decade, gene expression and deletion studies have revealed important functions of HDAC in adaptive responses of myocardial tissue to different types of injury, such as hypertrophy, ischemia, cell death, remodeling and fibrosis [5, 8]. Moreover, the development of specific small molecule inhibitors of HDACs allows for distinct studies of pharmacological interventions of HDAC function. HDAC inhibitors with a longer biological half time such as the class I HDAC inhibitor Entinostat (MS-275) are currently being used in clinical studies in patients with several types of hematological malignancies [9, 10] and may represent a significant therapeutic advance.

Recent evidence implies that several HDAC inhibitors could also be valuable tools to blunt pathological cardiac remodeling in the settings of pressure overload and ischemia/reperfusion [11–13]. In a recent study, Entinostat was shown to suppress hypoxia-dependent pulmonary hypertension and RV hypertrophy [14]. Here, we describe electrophysiologic effects of Entinostat in vivo in an experimental model of heart failure. However, due to the inherent biological differences between mouse and human hearts, study results in mice may have only a limited relevance to humans. Instead, the rabbit heart shares some physiological commonalities with the human heart, e.g. a similar ion channel expression pattern and similarities in myocardial action potential [15], and has therefore been successfully used for electrophysiological studies [16]. Using an in vivo model of heart failure, we studied the ability of the small molecule class I HDAC inhibitor Entinostat to prevent proarrhythmic effects of heart failure and structural remodeling associated with the development of heart failure.

## Methods

### Animals

All experimental protocols were approved by the local animal care committee (Landesamt für Natur, Umwelt und Verbraucherschutz Nordrhein-Westfalen) and conformed to the Guide for the Care and Use of Laboratory Animals published by the US National Institutes of Health (NIH Publication No.852–3, revised 1996).

### Induction of heart failure and administration of Entinostat

Adult female New Zealand white rabbits (weight 3.0–3.5 kg) were anesthetized intramuscular with Medetomidin (0.2 mg/kg), Midazolam (1 mg/kg) and Fentanyl (0.02 mg/kg). A pacemaker lead was implanted into the right ventricle via the right jugular vein and started stimulating with 400 beats/min 3 days later over a period of 10 days as recently described in detail [16]. The rabbits were examined echocardiographically at the first and last day of stimulation. Cycle length, left ventricular end-diastolic dimension and left ventricular end-systolic dimension were measured and the fractional shortening was calculated. The four treatment groups were compared in order to analyze the pharmacological effects of Entinostat in failing (i.e. rapidly stimulated) hearts: Sham rabbits (without rapid stimulation) treated with vehicle (DMSO) or Entinostat, respectively, and failing (i.e. rapidly stimulated) rabbits treated with DMSO or Entinostat, respectively. A total of 7 sham-operated rabbits and 6 heart failure rabbits were treated subcutaneously with Dimethylsulfoxide (DMSO) and served as controls, whereas 9 sham-operated rabbits and 6 heart failure rabbits were treated subcutaneously with Entinostat (0.1 g/d, LC Laboratories, Woburn, USA) over a period of 10 days. Entinostat is a benzamide HDAC inhibitor, which selectively inhibits class I HDACs 1 and 3. Entinostat effectively reduced hypoxia-induced pulmonary hypertension and reduced hypoxia-mediated RV hypertrophy in rats [14]. It has demonstrated efficacy in vitro and in vivo against a variety of tumors [9]. Entinostat has been shown to reduce myocardial damage in response to ischemia and reperfusion.

### Langendorff system

Following treatment with Entinostat (or DMSO, respectively) and rapid stimulation (or sham, respectively), animals were euthanized by anesthetic overdose (thiopental 100 mg/kg body weight i.v.) followed by cutting the cervical vessels. After removal of the pacemaker electrode, a thoracotomy was performed and the spontaneously beating heart was retrieved. Spontaneously beating hearts were attached to a vertical Langendorff apparatus (Hugo Sachs Elektronic, Medical Research Instrumentation, March-Hugstetten, Germany) as recently described [16–18] and retrogradely perfused with warm Krebs-Henseleit solution. A volume-conducted ECG was recorded by complete immersion of the heart into a bath of Krebs-Henseleit solution. Seven Monophasic action potential (MAP) catheters were evenly spread in a circular pattern around both ventricles and one MAP was recorded from the left endocardium. After placing the MAP catheters and the ECG and mechanical induction of complete AV block, the I_Kr_-blocking agent erythromycin (300 μM) (Inresa Arzneimittel GmbH, Freiburg, Germany) was infused just above the heart to create a proarrhythmic milieu and to better study the underlying mechanisms of arrhythmogenesis in heart failure [16]. The electrophysiology of 5 sham-operated rabbits and 5 heart failure rabbits treated with DMSO and 8 sham-operated rabbits and 4 heart failure rabbits treated with Entinostat were recorded at the Langendorff apparatus. Cycle length-dependence was investigated by pacing the hearts at cycle lengths between 900 and 300 ms. The MAP_90_ was measured as the average interval between the fastest MAP upstroke and 90% repolarization value. The QT-interval were observed and documented from stimulator recordings for each stimulated cycle length. Early afterdepolarisations (EAD) and polymorphic ventricular tachycardia resembling torsade de pointes were detected under low potassium concentration (1.5 μM) in spontaneously beating hearts.

### Immunohistochemistry

Tissue samples of Langendorff-perfused hearts (7 sham-operated rabbits and 6 heart failure rabbits treated with DMSO and 9 sham-operated rabbits and 6 heart failure rabbits treated with Entinostat) were fixed in 4% paraformaldehyde, processed and embedded in paraffin wax. The paraffin-fixed specimens were sliced into 6-μm-thick sections and stained with hematoxylin and eosin (HE) for general morphologic evaluation and Picrosirius red for detection of collagen deposits (fibrosis). Three sections of each rabbit heart were completely examined under a microscope with 4× objectives and the volume fraction of collagen was analyzed using the ImageJ 1.47 software.

### Preparations of protein lysates and Western blot analyses

Heart tissue was homogenized and lysed in RIPA buffer (Cell Signaling Technology) with 1 mmol/l PMSF for 30 min on ice and in an ultrasonic bath for 3 min. Proteins were separated by a sodium dodecyl-sulfate polyacrylamide gel and then transferred to a nitrocellulose membrane (Bio-Rad). Membranes were blocked with 5% nonfat milk in TBST and then incubated with the following primary antibodies: anti-acetylated histone H3 (anti-rabbit, 1:500, bs-3774R-Biotin, Bioss), anti-histone H3 (anti-rabbit, 1:500, sc-10,809, Santa Cruz Biotechnology), anti-Kv1.4 (anti-mouse, 1:500, 75–010, Antibodies Incorporated), anti-Kv1.5 (anti-mouse, 1:500, NBP2–12922, Novus Biologicals), anti-Erg (anti-rabbit, 1:1000, ARP35450_P050, avivasysbio), anti Cav1.3 (anti-mouse, 1:1000, mab60065, Covalab Biotechnology) and anti-Actin (anti-goat, 1:1000, sc-1616, Santa Cruz Biotechnology) at 4 °C over night. After an incubation for 1 h with secondary anti-rabbit (1:1000, P0448, Dako), anti-mouse (1:1000, P0260, Dako) or anti-goat (1:5000, sc-2020, Santa Cruz Biotechnology) the antigen-antibody complexes were visualized by enhanced chemiluminescence detection (ECL; Amersham Biosciences). The protein expression was quantified by Living Image Software (Caliper Life Sciences), and the value of each protein band was divided by the corresponding value of actin. The Blocking peptid (KCNH2 peptid, MFG42684, Aviva Systems Biology) was preincubated with a 1:1000 dilution of antibody in TBST at 4 °C over night. After centrifugation the same volume of 5% nonfat milk in TBST was added to the supernatant and incubated with the membrane at 4 °C over night.

### Statistical analysis

Quantitative analyses were performed using the Software Package for Statistical Science (SPSS for Windows; Version 22, SPSS Inc.; Chicago, IL). Normal distribution was assessed by the Shapiro-Wilk-Test, *p* > 0.05. Two-sample independent student’s t-tests were used to compare the means of two groups. One-way ANOVA was used to compare means of four samples. Differences with a *p* value of ≤0.05 were considered to be statistically significant.

## Results

### Alteration in failing hearts

**Fig. 1:**
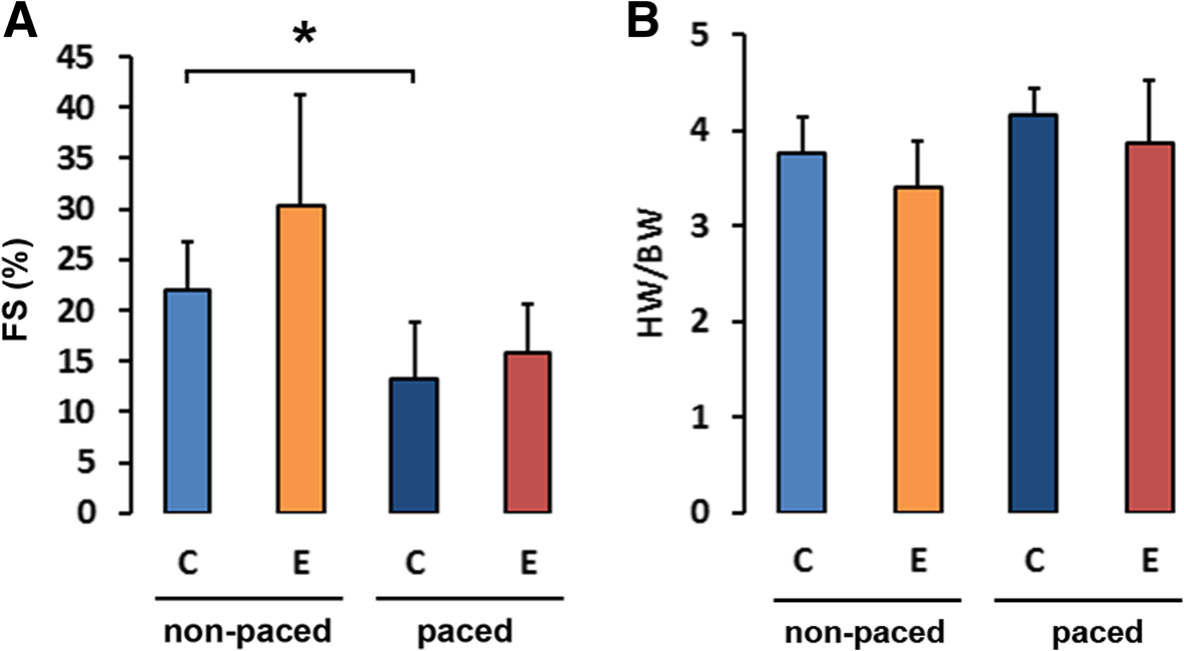
Effects of pacing on the fractional shorting and the heart weight in rabbits. **a** Two-dimensional echocardiography was carried out at the first and the last day of ventricular stimulation and fractional shorting (FS) were determined (One-way ANOVA *p* = 0.006, t-test (control hearts DMSO vs. failing hearts DMSO) *p* = 0.029 (*)). **b** To examine the development of hypertrophy in rabbit hearts the ratio of heart weight (HW) and body weight (BW) were determined in Entinostat treated (E) and vehicle treated rabbits (C) after 10 days of stimulation

**Fig. 2:**
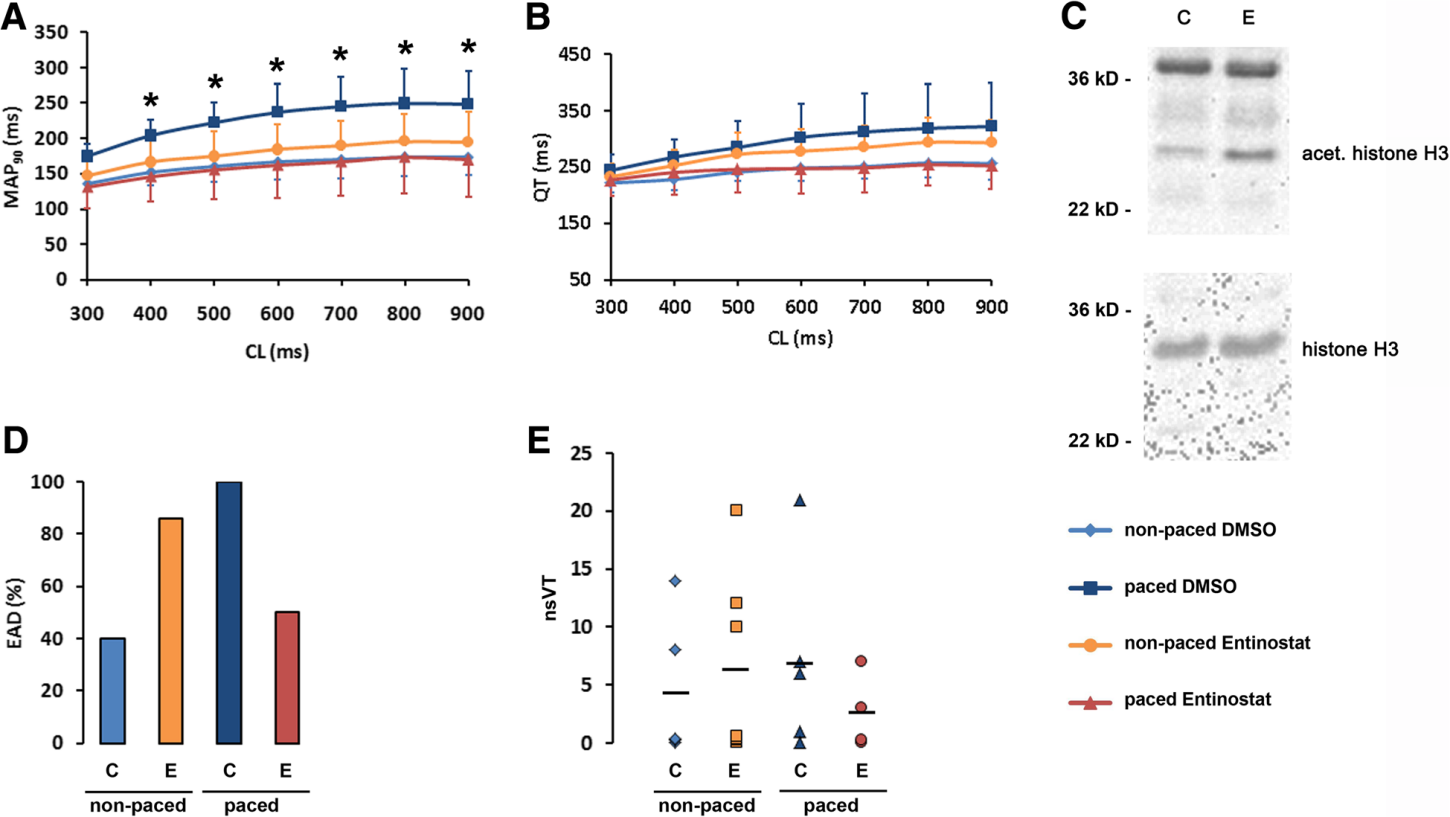
Effects of Entinostat on key electrophysiological parameters in control and rapidly paced rabbit hearts. **a** Cycle lengths-dependent effects on monophasic action potential (MAP_90_) in sham-operated hearts (*light blue*) and failing hearts (*dark blue*) treated with 0.5 ml/d DMSO and in sham-operated hearts (*orange*) and failing hearts (*red*) treated with 0.01 g/d Entinostat (One-way ANOVA p_(CL400–900)_ = 0.030, 0.034, 0.035, 0.041, 0.030, 0.027 (*)). **b** Cycle lengths-dependent effects on QT-interval in sham-operated rabbit hearts as compared to failing hearts. **c** A representative Western blot analysis demonstrating an increase of histone H3 acetylation in Entinostat (E) treated hearts compared to vehicle (C). **d** Proportion of rabbit hearts showing early afterdepolarizations (EADs) while the extracellular K^+^ concentration was lowered to 1.5 mM. **e** Number of non-sustained ventricular tachyarrhythmias (nsVT) per rabbit heart in the presence of low extracellular K^+^ concentration. On average, 7 episodes of non-sustained polymorphic tachycardias were observed in failing hearts treated with DMSO compared to 4 non-sustained ventricular tachyarrhythmia episodes in the non-stimulated control group and 2.5 non-sustained ventricular tachyarrhythmia episodes in failing hearts treated with Entinostat

The rabbits with induced heart failure became lethargic, developed ascites, pleural and pericardial effusion, peripheral oedema and developed an impairment of systolic left ventricular function during the course of 10 days. Two-dimensional echocardiography was carried out at the beginning and at the end of pacing and revealed a significant mean decrease in fractional shortening from 29.5 ± 10.5% (s.d.) to 16.4 ± 9.9% (s.d.) after 10 days of rapid pacing (Fig. 1a). Following ventricular pacing, the rabbit hearts did not display cardiac hypertrophy in this model (Fig. 1b). After pacemaker stimulation for 10 days, rabbit hearts were isolated and retrogradely perfused rabbits with heart failure displayed a significant MAP_90_ prolongation (from 173 ± 26 ms (sham) to 250 ± 41 ms in stimulated hearts; cycle length 900 ms; *p* < 0.05) (Fig. 2a) and a QT-interval prolongation compared to hearts from sham-operated rabbits (from 228 ± 20 (sham) to 268 ± 32 ms in stimulated hearts; cycle length 400 ms) (Fig. 2b).

### Electrophysiological effect of Entinostat in sham and failing hearts

Western blot analyses showed an increase of acetylated histone H3 protein in Entinostat treated hearts (Fig. 2c and Additional file 1: Figure S3) demonstrating that Entinostat efficiently inhibited class I HDACs in cardiac tissue. Electrophysiological measurements displayed that Entinostat treatment alone had no significant effect on MAP_90_ duration. However, treatment with Entinostat prevented the MAP_90_ prolongation observed in the failing hearts (from 250 ± 41 ms in stimulated hearts to 170 ± 53 ms in stimulated and treated hearts; cycle length 900 ms; *p* < 0.05) (Fig. 2a), suggesting that Entinostat prevented the proarrhythmic effect of ventricular tachypacing in failing hearts. Entinostat tended to result in a reduced mean QT-interval in failing rabbit hearts compared to sham rabbits (268 ± 32 ms in stimulated hearts to 240 ± 40 ms in stimulated and treated hearts; cycle length 400 ms) (Fig. 2b) even though this difference did not reach the level of statistical significance. Moreover, in accordance with prevention of MAP_90_ prolongation in failing hearts, Entinostat prevented the occurrence of EAD in failing rabbit hearts (Fig. 2d). At low K^+^ concentration, the number of non-sustained polymorphic tachycardias in rabbit hearts was heterogeneous. Nevertheless, Entinostat tended to reduce the number of non-sustained polymorphic tachycardias in failing hearts (Fig. 2e, Additional file 2: Figure S1A, B). Again, Entinostat prevented proarrhythmic effects of heart failure by reducing the occurrence of tachyarrhythmia episodes in rapidly stimulated, failing hearts. Of note, application of Entinostat caused an increase in polymorphic ventricular tachyarrhythmias in sham operated animals. This effect could possibly be attributed to a prolongation of the QT-interval by Entinostat application in sham hearts (Fig. 2b), while Entinostat application prevented a QT prolongation in failing hearts. Accordingly, EAD were prevented by Entinostat in failing hearts, while Entinostat treatment in sham hearts led to an increase of EAD (Fig. 2d).

### Effect of Entinostat on ion channel expression in sham and failing hearts

**Fig. 3:**
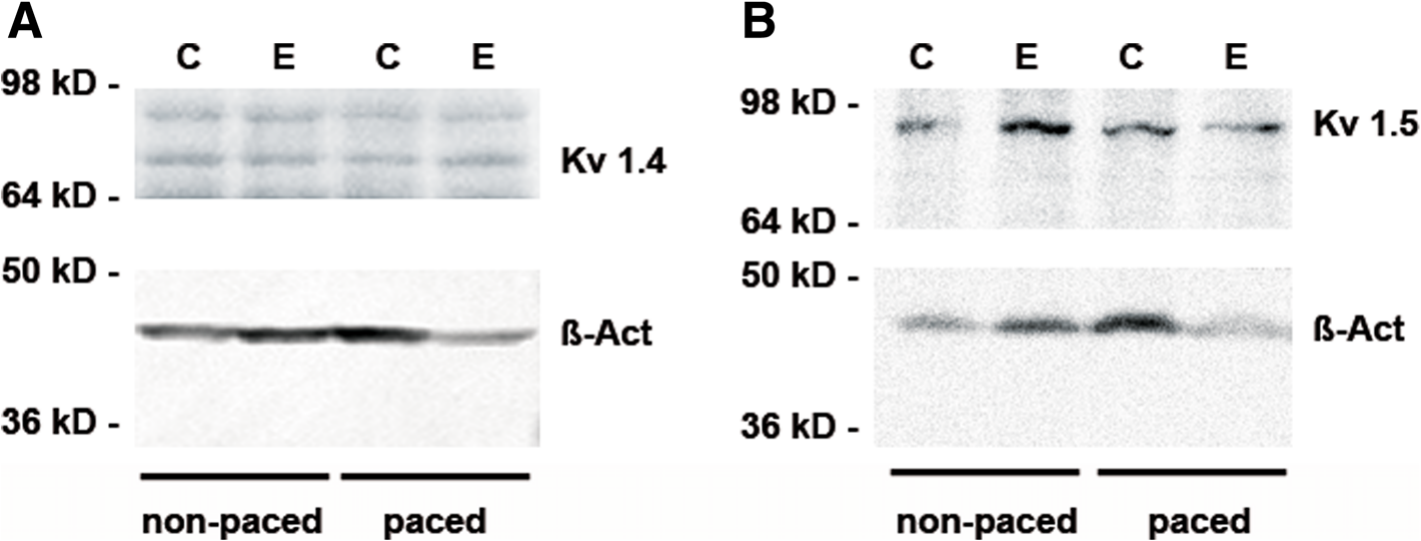
Effect of pacing and Entinostat treatment on the expression of potassium channels Kv1.4 and Kv1.5 in rabbit hearts. **a**, **b** Representative western blots showing protein expression of the potassium channels potassium voltage-gated channel subfamily A member 4 (Kv1.4, rabbit alpha subunit) and potassium voltage-gated channel subfamily A member 5 (Kv1.5) in rabbit hearts

**Fig. 4:**
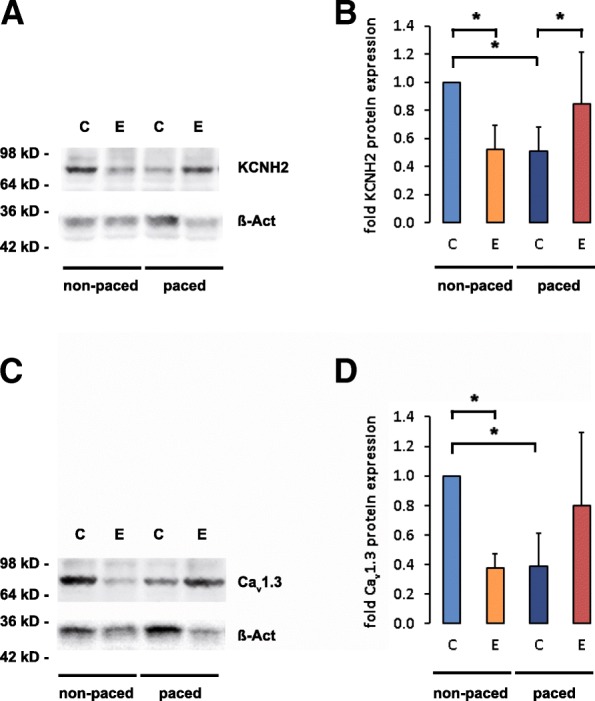
Effect of pacing and Entinostat treatment on the expression of the potassium channel KCNH2 and the calcium channel Cav1.3 in rabbit hearts. Representative western blots showing protein expression of the Potassium Voltage-Gated Channel, Subfamily H (Eag-Related), Member 2 (KCNH2) (*n* = 8, control DMSO vs. control Entinostat *p* < 0.001 (*), control hearts treated with DMSO vs. failing hearts treated with DMSO *p* < 0.001 (*), failing hearts treated with DMSO vs. failing hearts treated with Entinostat *p* = 0.043 (*)) (**a**, **b**) and the Ca^2+^ channel Cav1.3 (*n* = 4, control hearts treated with DMSO vs. control hearts treated with Entinostat *p* < 0.001 (*), control hearts treated with DMSO vs. failing hearts treated with DMSO *p* = 0.012 (*)) (**c**, **d**). The protein expression was quantified by Living Image Software. All western blots were normalized to DMSO not stimulated = 1

In order to decipher the mechanisms by which Entinostat changes MAP duration in failing hearts, we studied ion channel expression on the protein level. Since potassium channels play an important role in action potential duration and shape, we quantified the protein levels of potassium channels Kv1.4, Kv1.5 and KCNH2. No significant change in the expression level could be observed in case of Kv1.4 and Kv.1.5 (Fig. 3a, b, Additional file 1: Figure S3). Interestingly, KCNH2 and the Ca^2+^ L-type channel Cav1.3 demonstrated significant changes in protein expression depending on heart failure and Entinostat treatment (Fig. 4a–d, Additional file 3: Figure S2). KCNH2 and Cav1.3 were downregulated in response to pacing, and Entinostat was able to partly restore KCNH2 and Cav1.3 expression in failing hearts.

### Effects of Entinostat on fibrosis in vivo

**Fig. 5:**
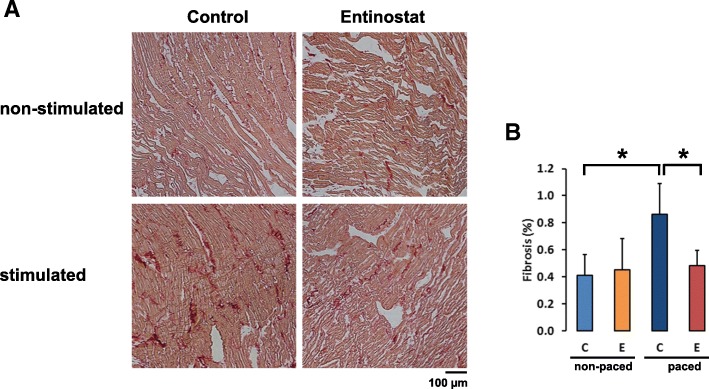
Effects of pacing and Entinostat treatment on the development of cardiac fibrosis in vivo. **a** Representative images of Picosirius Red staining of non-stimulated rabbit hearts and ventricular stimulated hearts treated with 0.5 ml/d DMSO (control) or 0.01 g/d Entinostat for 10 days. **b** The fibrotic areas were quantified by ImageJ software (*dark blue*: failing hearts treated with DMSO (*n* = 6); *light blue*: sham-operated hearts treated with DMSO (*n* = 7); *red*: failing hearts treated with Entinostat (*n* = 6) and *orange*: sham-operated hearts treated with Entinostat (*n* = 9) (control hearts treated with DMSO vs. failing hearts treated with DMSO *p* = 0.002, failing hearts treated with DMSO vs. failing hearts treated with Entinostat *p* = 0.003 (*))

In order to determine if structural changes underlie the observed electrophysiological alterations in addition to the effects on ion channel expression, rabbit hearts of all treatment groups were examined histologically. To assess the development of fibrosis in the heart, histological specimens from the left ventricle were analyzed with picrosirius red staining. In response to rapid pacing, rabbit hearts developed marked interstitial fibrosis. Treatment with Entinostat in non-paced hearts did not alter the degree of cardiac fibrosis. However, Entinostat was capable of reducing the formation of fibrosis in failing hearts (Fig. 5a, b).

## Discussion

The current study provides insight into the pharmacological effects of the class I HDAC inhibitor Entinostat on electrical and structural remodeling in heart failure. Class I HDACs are considered to mediate maladaptive pathways in response to cardiomyocyte stress and are assumed to have a negative impact on cardiac function. Accordingly, HDAC inhibitors have been shown to diminish or even reverse the cardiac remodeling process in several models of cardiomyopathy. In vivo*,* Entinostat treatment could prevent the development of cardiac fibrosis in response to rapid ventricular stimulation.

Rapid ventricular stimulation caused a significant elevation of monophasic action potential duration compared to sham hearts while treatment with Entinostat could partially abolish this effect. In failing hearts, Entinostat could partially restore the reduction of Cav1.3 and KCNH2 expression.

While our model of pacing induced heart failure did not result in the development of marked cardiac hypertrophy, different HDAC inhibitors have been shown to be able to reduce cardiac hypertrophy. Specifically, the pan HDAC inhibitor TSA blunted cardiac hypertrophy and improved systolic function in a pressure-overload transverse aortic constriction model in mice [7] and TSA was even capable of reversing pre-existing hypertrophy [19]. In a rat infarct model, the HDAC inhibitors valproic acid (VPA) reduced cardiomyocyte hypertrophy and collagen deposition of the infarcted left ventricle and preserved systolic function [20]. However, a preservation of cardiac systolic function could not be demonstrated in our model. This observation can possibly be explained by the specific conditions of rapid ventricular pacing that subjects the heart to a different type of stress compared to other models of heart failure.

In fact, pressure overload induced heart failure is characterized by an adaptive increase in the ratio of LV wall thickness to chamber radius thereby partly compensating the increase in afterload against which the myocardium shortens during systole. The resulting concentric hypertrophy is associated with profound changes in gene expression pattern resembling that of fetal hearts [21]. Histone deacetylase inhibitors have shown to be able to reverse some of these gene expression changes suggesting that they may have a therapeutic potential in cardiac hypertrophy and heart failure. Interestingly, KCNH2 and Cav1.3 protein expression levels could be partially restored by Entinostat in failing, stimulated hearts. On the contrary, heart failure in our model is caused by tachycardia in combination with asynchronic LV contraction. In this model, rapid pacing typically leads to LV chamber dilatation and reduced wall thickness. Thus, the different pathomechanisms leading to heart failure in both models might explain why Entinostat did not efficiently preserve LV function during rapid pacing despite its antiproliferative and antifibrotic effects.

In our model, long term rapid ventricular pacing of rabbit hearts caused a significant increase of MAP duration, more EAD and more non sustained polymorphic ventricular tachycardias compared to sham hearts [16]. Classically, these effects have been attributed to a down-regulation of potassium channels, resulting in a statistically noticeable prolongation of the QT-intervals and in extended monophasic action potentials as a consequence of a prolonged repolarization process [22]. Like in the context of heart failure, an excessive prolongation of action potential duration leading to EAD [23] and an increase of spatial dispersion of repolarization can act as a substrate for the occurrence of proarrhythmia [24]. Here, we show that treatment with Entinostat could partially prevent these effects by prevention of MAP prolongation observed in failing hearts. Consistently, Entinostat was able to partially restore KCNH2 ion channel expression downregulated in rabbit hearts with pacing induced heart failure. Thus, Entinostat likely contributes to a reduction of spatial dispersion of repolarization and causes a stabilization of repolarization and prevention of ventricular tachyarrhythmias. Correspondingly, EAD were reduced by half after administration of Entinostat in failing hearts, and that may account for the lack of proarrhythmia in failing hearts treated with Entinostat. However, in non-paced, structurally normal hearts, Entinostat paradoxically displayed a weak tendency to prolong QT-interval in association with more EADs and more non-sustained ventricular tachyarrhythmias. Therefore, the pharmaceutical effect of Entinostat in healthy hearts with regard to QT interval duration and potassium channel activity seems to be complex. In clinical trials, the HDAC inhibitors Vorinostat and Romidepsin have been demonstrated to cause a prolongation of the QT-interval [25]. In patients with metastatic neuroendocrine tumors, the HDAC inhibitor Depsipeptide FR901228 has been associated with QT-interval prolongation and ventricular tachycardias [26]. In a model of ischemia, however, Entinostat was able to confer protection from myocardial damage by ischemia/reperfusion. Interestingly, this effect was dependent on mitochondridal HDAC1 [27].

Myocardial potassium channel expression is subject of a complex regulation on the transcriptional, post-transcriptional, post-translational and epigenetic level [28]. Potassium channels play an important role in action potential duration and shape. The potassium channels Kv1.4, Kv1.5 and KCNH2 are important molecular components of I_to_, I_Kur_ and I_Kr_, respectively [29]. Since epigenetic mechanisms determining cardiac potassium and calcium channel activity have not yet been well characterized, one might speculate that the effects of class I HDAC inhibition by Entinostat on potassium and calcium channel activity critically depend on transcription factors and key regulators of potassium channel expression and activity that are induced or repressed as a result of the development of cardiac failure induced by rapid ventricular pacing. Data on ERG expression in failing hearts has been inconsistent, few studies report a reduction of ERG expression in heart failure [30]. Li et al. did not detect significant differences in mRNA ERG expression in the left ventricles among rabbits with heart failure [31]. In our study, the downregulation of the ERG ion channel KCNH2 in failing rabbit hearts might have contributed to the observed prolongation of MAPs. In contrast to our results, the expression of I_to_ subunit Kv1.4 mRNA was significantly down-regulated in rabbits with heart failure [31]. Changes in Kv1.5 expression have been variable related to differences in underlying heart disease [32]. In fact, a different expression of selected potassium channel species (Kv1.4 and Kv1.5) could not be confirmed in our study (Fig. 3). This finding is probably related to different stimulation frequency and duration. In addition, other mechanisms including posttranslational channel protein modification may play a role. Hence, further studies will be necessary to clarify these issues. Interestingly, HDAC inhibitors showed several cardiac side effects including atrial fibrillation and QT prolongation in different clinical trials.

Finally, histological analysis of tissue specimen of rabbit hearts revealed that Entinostat could prevent the development of cardiac fibrosis in response to rapid ventricular stimulation. Several studies in animal models imply that HDAC inhibition have beneficial effects on disease states associated with pathological fibrosis. Class I HDAC inhibition retards fibrosis formation in acute myocardial infarction settings and suppresses angiotensin II-mediated cardiac fibrosis [33]. In general, fibrosis has significant consequences for cardiac function, since progressive increases in extracellular matrix synthesis and deposition result in increased mechanical stiffness and contribute to systolic dysfunction. Depending on the stage of heart failure, myofibroblasts are highly localized to sites of injury where synthesis and deposition of collagen promotes scar formation and fibrosis [34]. Specifically, progressive cardiac fibrosis fosters the development of malignant arrhythmias based on local reentry. Consequently, the prevention of cardiac fibrosis might therefore additionally protect the heart from the occurrence of arrhythmias. Thus, Entinostat seems to be a promising candidate for the prevention of electrical and structural remodeling in heart failure.

### Limitations

Cardiac remodeling is a complex process. Besides cardiac fibrosis, additional issues such as mechanical stretch, hormonal influences, and local and systemic inflammation play important roles but were not addressed in our studies. In fact, the complex interplay between cardiomyocytes, cardiac fibroblasts, and immune cells in the context of pharmaceutical HDAC inhibition warrants further investigation. Moreover, key electrophysiological parameters were measured in isolated rabbit hearts. Direct comparison to human heart failure is therefore limited. In fact, the rabbit heart might response differently to rapid pacing in comparison to human hearts. Moreover, despite a certain similarity in action potential shape and duration between human and rabbit hearts, repolarization in rabbit cardiomyocytes is mainly influenced by I_Kr_, whereas the cells express little I_Ks_. Acute effects of Entinostat on ventricular electrophysiology were not assessed, and ion channel measurements were not performed. Therefore, in order to overcome the inherent limitations of the model used in this study, further investigations will be necessary to clarify the pharmaceutical potential of Entinostat in the setting of heart failure.

## Additional files

Additional file 1: **Figure S3.** Representative western blots of Kv1.4 and Kv1.5 and protein expression quantification. (A) Whole western blots of Fig. 3 are displayed. The Kv1.4 alpha subunit in rabbits has a molecular weight of 71.9 kD. So we clarified the band above the 64 kD marker band as Kv1.4. The rabbit potassium channel Kv1.5 (B) has a molecular weight of 65.5 kD. Therefore the band between 64 kD and 98 kD was identified as Kv1.5. (C-D) Quantification of Kv1.4 (C) and Kv1.5 (D) protein expression. The protein expression was quantified by Living Image Software. All western blots were normalized to DMSO not stimulated = 1. n.s. indicates no significant difference. (E) Quantification of acetylated H3 to total H3 protein expression. * *p* < 0.05. (PDF 13066 kb)

Additional file 2: **Figure S1.** Representative example of after-depolarizations and torsade de pointes in rabbit hearts at the Langendorff system. Example of after-depolarizations and torsade de pointes in a sham-operated (A) and in a failing heart (B) after 10 days of ventricular pacing during bradycardia (atrioventricular-block) and hypokalemia in an isolated Langendorff-perfused rabbit heart. Monophasic action potential-recordings (MAP 1 = left ventricular base anterior; MAP 2 = right ventricular apex anterolateral; MAP 3 = right ventricular base anterolateral; MAP 4 = left ventricular base posterior; MAP 5 = left ventricular between base and apex posterolateral; MAP 6 = left ventricular between base and apex lateral; MAP 7 = left ventricular apex; MAP 8 = left endocardium apex) and left ventricular pressure (LV-pressure). (TIF 25516 kb)

Additional file 3: **Figure S2.** Western blot with KCNH2 antibody and blocking peptide. A highly dense band between 98 kD and 64 kD was detected in rabbit heart when sample was immunoblotted with KCNH2 antibody not exposed to blocking peptide (−). These band was absent from immunoblot blocked by preincubation of antibody with antigenic peptide (+). (TIF 1028 kb)

## Abbreviations

DMSO: Dimethylsulfoxide
EAD: Early afterdepolarisations
FS: Fractional shortening
HDAC: Histone deacetylase
MAP: Monophasic action potential
TdP: Torsade de pointes
TSA: Trichostatin A
