# Supplementary figures and images for: Histone deacetylase inhibition by Entinostat for the prevention of electrical and structural remodeling in heart failure
Source: BMC Pharmacol Toxicol. 2019 Mar 6;20:16. doi: 10.1186/s40360-019-0294-x (PMC6404297; doi:10.1186/s40360-019-0294-x)

**A**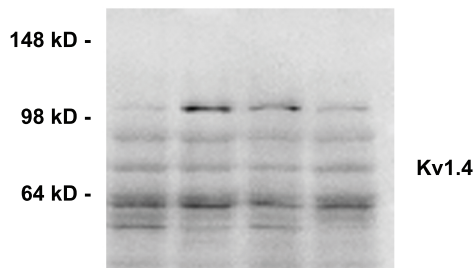**B**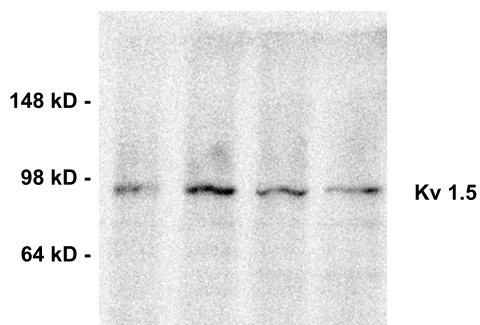**C**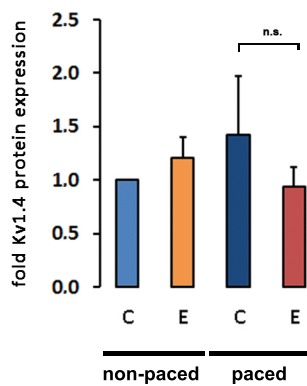**D**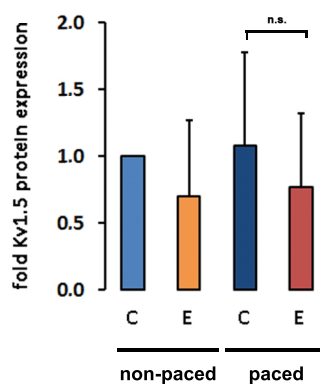**E**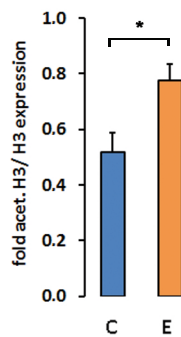

Supplement: Supplementary file 1 — Figure S3. Representative western blots of Kv1.4 and Kv1.5 and protein expression quantification. (A) Whole western blots of Fig. 3 are displayed. The Kv1.4 alpha subunit in rabbits has a molecular weight of 71.9 kD. So we clarified the band above the 64 kD marker band as Kv1.4. The rabbit potassium channel Kv1.5 (B) has a molecular weight of 65.5 kD. Therefore the band between 64 kD and 98 kD was identified as Kv1.5. (C-D) Quantification of Kv1.4 (C) and Kv1.5 (D) protein expression. The protein expression was quantified by Living Image Software. All western blots were normalized to DMSO not stimulated = 1. n.s. indicates no significant difference. (E) Quantification of acetylated H3 to total H3 protein expression. * p < 0.05. (PDF 13066 kb) [file 40360_2019_294_MOESM1_ESM.pdf]

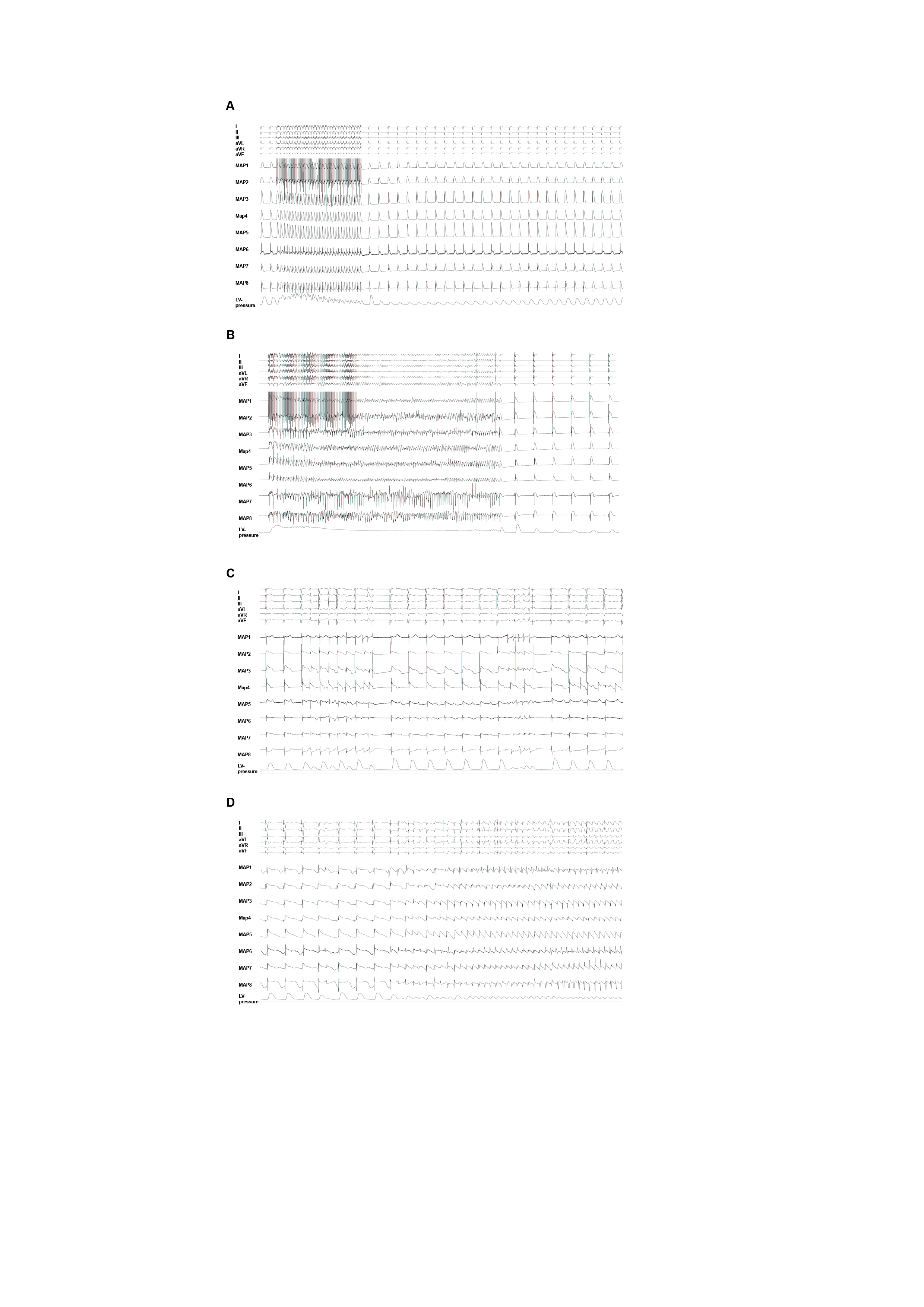

Supplement: Supplementary file 2 — Figure S1. Representative example of after-depolarizations and torsade de pointes in rabbit hearts at the Langendorff system. Example of after-depolarizations and torsade de pointes in a sham-operated (A) and in a failing heart (B) after 10 days of ventricular pacing during bradycardia (atrioventricular-block) and hypokalemia in an isolated Langendorff-perfused rabbit heart. Monophasic action potential-recordings (MAP 1 = left ventricular base anterior; MAP 2 = right ventricular apex anterolateral; MAP 3 = right ventricular base anterolateral; MAP 4 = left ventricular base posterior; MAP 5 = left ventricular between base and apex posterolateral; MAP 6 = left ventricular between base and apex lateral; MAP 7 = left ventricular apex; MAP 8 = left endocardium apex) and left ventricular pressure (LV-pressure). (TIF 25516 kb) [file 40360_2019_294_MOESM2_ESM.tif]

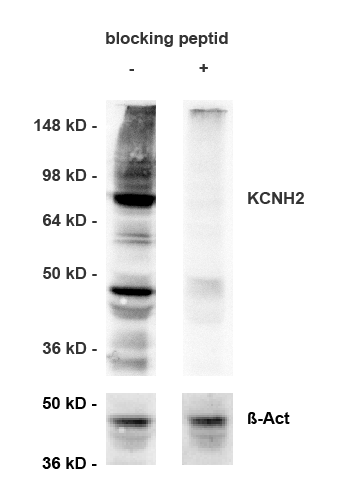

Supplement: Supplementary file 3 — Figure S2. Western blot with KCNH2 antibody and blocking peptide. A highly dense band between 98 kD and 64 kD was detected in rabbit heart when sample was immunoblotted with KCNH2 antibody not exposed to blocking peptide (−). These band was absent from immunoblot blocked by preincubation of antibody with antigenic peptide (+). (TIF 1028 kb) [file 40360_2019_294_MOESM3_ESM.tif]
